# A movable type bioelectronics printing technology for modular fabrication of biosensors

**DOI:** 10.1038/s41598-021-01741-1

**Published:** 2021-11-16

**Authors:** Muqun Yang, Mingyang Liu, Jing Cheng, Han Wang

**Affiliations:** 1 Precision Medicine and Healthcare Research Center, Tsinghua-Berkeley Shenzhen Institute (TBSI), Tsinghua University, Shenzhen, 518055 China; 2 Department of Biomedical Engineering, School of Medicine, Tsinghua University, Beijing, 100084 China

**Keywords:** Biomedical engineering, Lab-on-a-chip

## Abstract

Biosensors have been widely used in various fields such as food industry, environmental testing and medical testing for their high sensitivity. However, current fabrication methods of biosensors, such as screen printing, micro fabrication and 3D printing suffer from complex procedures, requirement of cleanroom facility and limited fabrication materials, which significantly restrict the development and utilization of biosensors. Here, we propose a movable type bioelectronics printing method for the fabrication of biosensors by directly transferring bioelectronic materials onto various substrates using pre-fabricated molds. This simple, low-cost, yet robust method facilitates on-demand printing of master molds of partial or complete circuits on both rigid or flexible substrates. With this method, bioactive materials such as enzymes can be directly transferred onto substrates together with other electronic components, without complex modification after electrode fabrication using conventional methods. For demonstration, a dual-channel flexible electrochemical biosensor was fabricated by the movable type bioelectronics printing method for continuous monitoring of glucose and lactate. The movable type bioelectronics printing technology holds advantages of repeatability, flexibility and low cost for fabrication of biosensors on rigid and flexible substrates, as well as direct transfer printing of bioactive materials, which greatly promotes small-scale production of biosensors.

## Introduction

Biosensors are analytical devices that combine biological components with physicochemical detectors. Due to advantages in high sensitivity and capability of automation and integration, biosensors have been widely used in various fields such as food industry^1,2^, environmental testing^2,3^ and medical testing^4,5^. A biosensor typically consists of a bio-receptor, a transducer that converts biological signals to physical signals such as optical or electrical signals, and electronic systems, which combines technologies from life science, analytical chemistry, engineering, material science and information technology, etc^6,7^. In recent years, considerable efforts have been devoted to identify alternative materials^7^, structures^8^ and modification methods^9^ for the improvement of performance in the sensitivity, linear range and limit of detection of biosensors. However, the complex procedures of biosensor fabrication restrict its development and utilization.

At present, there are three representative technologies to fabricate bioelectronic sensors. Screen printing is a printing method that forces the liquid paste through a mask by a squeegee to form a pattern onto the substrate surface, which has been widely used owing to the easiness in fabrication on an industrial scale^10,11^. However, one noteworthy limitation of the screen-printing fabrication method resides in its requirement of skilled printers and its high cost on small-scale production. Indeed, screen printing has largely been confined to flat surfaces, which may also restrict the potential utility of the method in the biosensors fields^12^. Micro fabrication methods offer the possibility for highly reproducible mass fabrication of biosensor with complex geometries and functionalities of through techniques such as film deposition, photolithography, etching, bonding and molecular self-assembly. These biosensors are widely applied in sweat analysis^13,14^, tear analysis^15,16^ and saliva analysis^17,18^. However, the microfabrication method is restricted by its complex processing procedures and requirement of cumbersome machines and cleanroom. Moreover, 3D printing is another available fabrication method referring to a variety of processes in which photo-sensitive materials are deposited, joined or solidified under computer control to produce a three-dimensional object, where materials like plastics, liquids or powder grains are added together layer by layer^19,20^. But it suffers from the limitation of available printing materials. Besides, all of these methods cannot realize direct fabrication and functionalization with bioactive materials and thus reduce the efficiency of research and the development of biosensors. Therefore, developing a method for small-scale production of biosensors that can simplify the fabrication and functionalization process is in urgent need.

Movable type printing is a system of printing with a long history, that uses movable components to reproduce the elements of a document usually on the medium like papers. The general procedure of movable type printing consists of the following steps: making a reverse style of a single character, picking out words according to the manuscript, arranging the words in the font plate and finally printing with ink. After printing, the typeface could be recycled for use in the next typesetting^21^. Inspired by this work, we propose a fabrication method for biosensors, which could benefit from simple fabrication and flexible assembly of master molds of bioelectronic components, and direct preparation of bioactive materials without complex surface modification. Besides, simultaneous detection of multiple metabolites is of great importance. Diabetic patients, for instance, might also suffer from many complications like lactic acidosis, which is a dangerous disease with extremely high fatality. There are few reports that could realize simultaneous detection of both lactate and glucose^22^. Lin et al. developed a microfluidic chip-based electrochemical system by micro-fabrication for simultaneous monitoring of glucose, lactate, and ascorbate in rat brain. However, this method suffered from complex processing procedures and complicated system construction^23^. Here, we present a movable type bioelectronics printing technology through the procedures of biosensor design and master mold preparation, transfer printing and post-transfer treatment. This movable type bioelectronics printing technology holds advantages in repeatability, easy modification and assembly for fabrication of biosensors, as well as direct fabrication with bioactive materials on both rigid and flexible substrates. To demonstrate the capability and performance of the fabrication method, we developed a dual-channel electrochemical biosensor for the simultaneous monitoring of lactate and glucose to help manage the concentration levels of these metabolites and avoid lactic acidosis. We envisage the movable type bioelectronics printing technology could help promote prototyping and small-scale production of biosensors.

## Methods

### Materials

Chitosan (50,000–190,000 Da), Nafion, glucose, lactate acid and glutaraldehyde solution (50% in H_2_O) were all purchased from Sigma (Steinheim, Germany). Glucose oxidase (10 units/mg) and lactate oxidase (36 units/mg) were purchased from Yuanye Bio-Technology Corporation (Shanghai, China). Polydimethylsiloxane (PDMS, Sylgard 184, Dow Corning Corporation, Midland, MI) was prepared by mixing the pre-polymer and curing agent at a ratio of 10:1 (by weight). The Ag conductive paste and carbon paste were purchased from Ausbond Corporation (Silver paste 3813 and carbon paste A528, Ausbond Corporation, Shenzhen, China). Phosphate buffer solution (pH 7.2–7.4) was from Solarbio (Beijing, China). Polyethylene terephthalate (PET) was purchased from Suzhou Gule Packaging Materials Company (Suzhou, China). All other chemicals used in this work were of analytical grade. Double distilled water was used throughout the experiments. All mold patterns were initially designed with a computer aided design software and printed with a light curing printer (CR-10, Shenzhen Creality 3D Technology Corporation, Shenzhen, China). All electrochemical measurements were performed on an electrochemical workstation (CHI660E, Chenhua Corporation, Shanghai, China).

### The mechanism of the movable type bioelectronics printing technology

**Figure 1:**
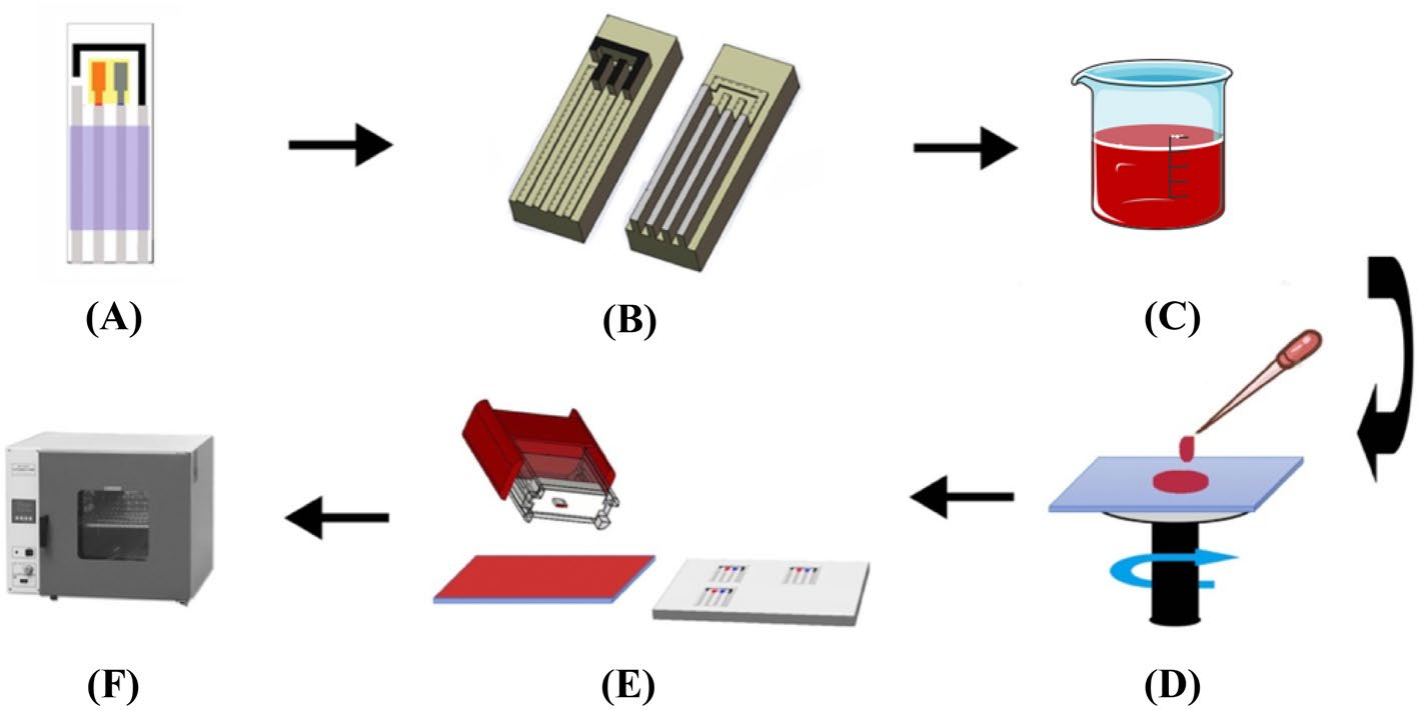
Illustration detailing the steps involved in the movable type bioelectronics printing method including **(A)** biosensor design, **(B)** mold design and fabrication, **(C)** bioelectronic material preparation, **(D)** spin-coating, **(E)** transfer printing and **(F)** post-transfer treatment with an oven for drying.

In this work, we propose a modular transfer printing technology as an alternative to conventional methods for biosensor fabrication (see Fig. 1). The proposed movable type bioelectronics printing technology is based on contact printing, which is a category of printing technology using master molds to transfer the patterns on the molds to the substrates. In our transfer printing technology, all the molds were designed and fabricated according to the requirement of different functional components of biosensors. Then a variety of bioelectronic materials can be used to prepare the pastes, such as conductive carbon paste, Ag/AgCl paste and insulation paste, etc., which were spun on a spin coating machine to form a thin layer. And then the pastes were stuck to the molds, transfer printed onto the substrates (see Fig. S1) and dried in the ventilating oven at 40 °C. This movable type bioelectronics printing technology facilitates flexible manufacturing using both individual molds and assembled molds to produce partial or complete bioelectronic devices. On one hand, the individual molds of different circuit components such as resistors, capacitors, and wireless communication coil, etc., can be used to directly transfer print partial circuits on both flexible and rigid substrates. On the other hand, the individual molds can be freely combined on-demand to print complete bioelectronic circuits at one time. As a demonstration of the versatility of this fabrication technique, in this work complete electrochemical sensor patterns were printed onto different substrates such as silicon wafer, PET, glass and human skin.

### Characterization and optimization of the transfer printed electrodes

To characterize the printed electrodes on rigid and flexible substrates based on the modular transfer printing technology, we studied the resistance change of printed electrodes with various thickness and bending angles. As the spin-coating speeds increased, bioelectronic materials on the substrates would become thinner and thus the resistance of the printed patterns would be higher. So we used printed electrodes as an example and studied the resistance change with spin-coating speeds and stability after bending. The electrodes with 5, 10, 15 mm-length were printed onto rigid and flexible substrates via two steps: at first spin-coating the conductive paste with various spin-coating speeds as shown in Table S1, secondly transfer printing onto rigid (glass) and flexible (PDMS) substrates with the molds. After dried at the room temperature, the resistance and thickness of all the electrodes were measured with a Fluke multimeter (Fluke 17B + , Fluke Corporation, Elfrid Payton, WA) and a stylus profiler (P-7 stylus profiler, KLA-Tencor Corporation, San Francisco, CA). For the flexible electrodes printed on PDMS, resistance change characteristics at different bending angles were studied by tuning the substrate from 10$^\circ$ to 130$^\circ$ with an interval of 20$^\circ$, and then the resistances were measured 4 times at each angle. The resolution of the printed electrodes was also measured by microscopy (QJY-9001, Shenzhen Industrial Vision Co., Ltd, Shenzhen, China).

### Fabrication of a dual-channel electrochemical biosensor

**Figure 2:**
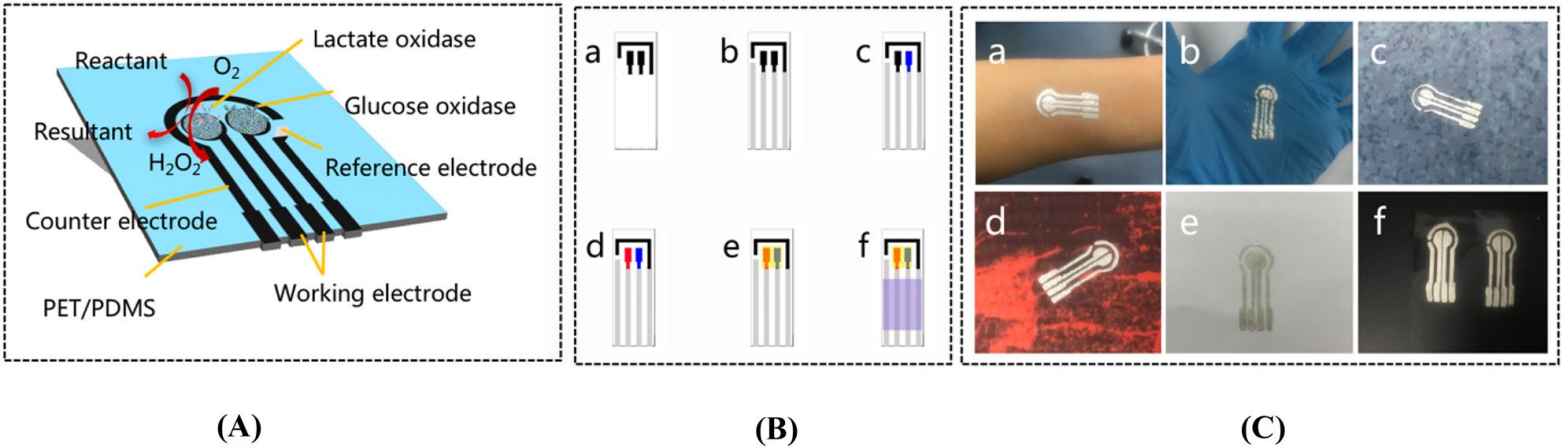
**(A)** Design and working mechanism of a dual-channel electrochemical biosensor for detection of lactate and glucose. The dual-channel biosensor consists of two working electrodes, one counter electrode and one reference electrode. Lactate oxidase and glucose oxidase are immobilized on separate working electrodes. **(B)** Step-by-step schematic diagram of a printed dual-channel biosensor using movable type bioelectronics printing technology. (a) transfer printing of the counter electrode and the working electrode layer with conductive carbon paste; (b) transfer printing of connecting wires and the reference electrode layer with Ag/AgCl paste; (c) and (d) transfer printing of lactate oxidase and glucose oxidase on their respective working electrode; (e) transfer printing of the protective Nafion layer for enzymes; (f) transfer printing of the insulator layer. **(C)** Fabricated dual-channel electrochemical biosensor on a variety of substrates including (a) epidermis, (b) gloves, (c) rough rubber, (d) sportswear, (e) papers and (f) PET respectively.

Lactate metabolism dysfunctiont is a common complication of diabetes, which would cause lactate accumulation and thus result in diabetic lactic acidosis with high mortality^24–26^. Thus, developing a dual-channel biosensor could help manage the concentration levels of glucose and lactate at the same time, preventing dangerous complications such as hypoglycemia and diabetic lactic acidosis. However, most of reported works on diabetic management can only measure the change of glucose level, partly due to the difficulty of complex fabrication and modification of dual-channel biosensors. In this work, we utilized the movable type bioelectronics printing technology to fabricate a dual-channel biosensor for glucose and lactate monitoring. This transfer printing method enabled direct fabrication and functionalization of the dual-channel electrochemical biosensor using lactate oxidase and glucose oxidase for detection of both lactate and glucose concentration levels. The mechanism of the dual-channel amperometric biosensor is based on enzymatic electrochemical reactions where lactate and glucose are oxidized (Fig. 2A). The general equations of the dual-channel amperometric biosensor are given below:

1 $$Glucose+{O}_{2}\stackrel{ GOx }{\to }Gluconate+{H}_{2}{O}_{2}$$

2 $${Lactic \,\, acid+O}_{2}\stackrel{ LOx }{\to }Pyruvate+{H}_{2}{O}_{2}$$

$GOx$ refers to glucose oxidase and $LOx$ refers to lactate oxidase. Then the produced H_2_O_2_ decomposes on the surface of the electrodes as the following equation shows and the corresponding current is detected:

3 $${O}_{2}+4{e}^{-}+4{H}^{+}\stackrel{ }{\to }2{H}_{2}{O}_{2}$$

Figure 2B shows the step-by-step process of fabricating the dual-channel biosensor, without complex functionalization with enzymes. The printing of the dual-channel biosensor contains these steps: Transfer printing counter and working electrode layers with conductive carbon paste, wires and reference electrode layer with Ag/AgCl paste; Then transfer printing bioactive materials including lactate oxidase and glucose oxidase solutions, respectively. The Chitosan/CNTs solution was prepared by adding 5 mg amino-functionalized carbon nanotubes into a 2 mL chitosan solution (0.2% wt) with ultrasonic mixing for 10 min to achieve complete dispersion. Then 1 mg lactate oxidase was added to the Chitosan/CNTs solution (50 μL) to obtain Lactate oxidase/Chitosan/CNTs solution (20 mg/mL for lactate oxidase). And Glucose/Chitosan/CNTs solution (10 mg/mL for glucose oxidase) was prepared using the same procedure as lactate oxidase/chitosan/CNTs solution. Finally, a protective Nafion (5% in water) layer for enzymes and an insulator layer (PDMS) were completed. With these printing procedures, we realized direct fabrication and functionalization with bioactive materials on PET substrates.

### Electrochemical characterization of the dual-channel biosensor

Differential pulse voltammetry (DPV), cyclic voltammetry and amperometric i–t curve are common methods for electrochemical analysis. DPV is a sensitive voltammetric technique that involves applying staircase amplitude potential pulses on a linear ramp potential. However, this method is not intuitive to detect the concentration of target analytes, and the data processing is inconvenient. In our biosensors, we mainly applied the amperometric i–t curve method which could directly and quickly reflect changes of response currents versus time. Cyclic voltammetry studies were conducted with the dual-channel biosensor using a CHI660E electrochemical workstation. A comparison test between an enzyme modified biosensor and an unmodified biosensor in 100 μL testing solution mixed with lactate (300 μM) and glucose (300 μM) was carried out. To study the characteristics of the dual-channel biosensor, cyclic voltammograms were recorded by injecting 100 μL potassium ferricyanide solution (0.01 M) into the working electrode at scanning rates ranging from 20 to 160 mV/s with a 20 mV interval. The potential range during cyclic voltammetry tests was between −0.30 and 0.50 V. To further evaluate the performance of the dual-channel biosensor for lactate and glucose detection, amperometric i–t curves were recorded after injecting 100 μL test samples containing lactate or glucose into the working electrode at room temperature (25˚C). The concentration of lactate samples varied from 0 to 1000 μM and the concentration of glucose samples varied from 0 to 1000 μM. After each measurement, the working electrode was cleaned three times with DI water. The potential of amperometric i-t tests was set at 0.30 V and the testing time was set to 100 s.

### Specificity and stability studies of the dual-channel biosensor

Firstly, to study the specificity of the dual-channel biosensor, amperometric i–t measurement of the biosensor was carried out by adding different interferents such as uric acid at 50 μM, and ascorbic acid at 0.1 mM, respectively, into 100 μL PBS solution. The concentration of each interferent was chosen based on the average concentration level in healthy human blood. Secondly, to evaluate the stability of the dual-channel biosensor, after testing with lactate and glucose as described in the previous session, the dual-channel biosensor was washed by PBS buffer, dried and stored in a refrigerator at 4 ˚C for around one week. Then the biosensor was retrieved and the performance was investigated by amperometric i–t test every week for a total of 4 weeks with lactate (300 μM) and glucose (300 μM) samples. The potential of amperometric i–t tests was set at 0.30 V and the testing time was set to 100 s. The measured currents of the dual-channel biosensor before and after storage were compared.

### Analysis of human serum samples

To investigate the feasibility of the dual-channel biosensor for measurement of lactate and glucose in biological fluids, serum samples from healthy human volunteers were diluted by PBS and measured by our biosensor, and compared with the FDA-approved electrochemical glucose analyzer (Accu-Chek Advantage, Roche Diagnostics Corporation, Basel, Switzerland) and lactate analyzer (Lactate Scout 4, EKF Diagnostics Corporation, Barleben, Germany). The serological samples were collected in anticoagulant tubes and used immediately after collection. All experimental protocols were approved by the Ethics Review Committee of Tsinghua University (Beijing, China). All methods were performed in accordance with the relevant guidelines and regulations by the Ethics Review Committee of Tsinghua University (Beijing, China) and informed consent was obtained from all participants.

## Results and discussion

In this work, we proposed a modular transfer printing technology for prototyping and small-scale fabrication of biosensors. The movable type bioelectronics printing technology holds advantages in flexible manufacturing using individual molds as well as assembled molds for patterning different combinations of bioelectronic components on-demand. Besides, this movable type bioelectronics printing technology enables transfer printing of bioelectronic patterns on a variety of rigid or flexible substrates. Figure 2C illustrates transfer printing of a dual-channel electrochemical biosensor on different substrates such as epidermis, gloves, rough rubber, sportswear, papers and PET, respectively. This demonstrated the good applicability of the movable type bioelectronics printing technology using various substrates including unconventional non-planar and curvilinear substrates.

**Figure 3:**
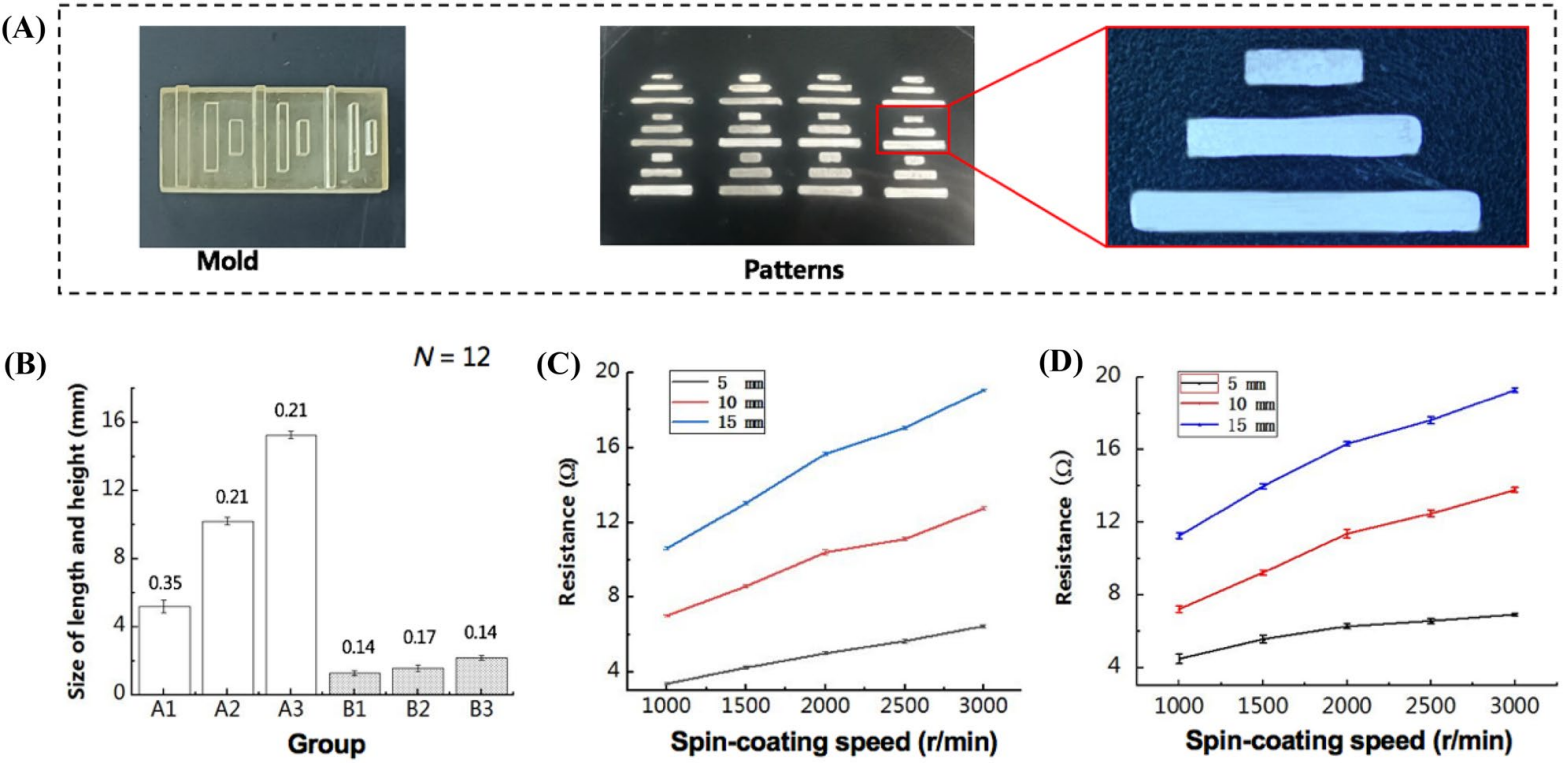
Resolution tests of the transfer printed electrodes. **(A)** Photographs of electrode molds by a light curing printer and the printed electrode patterns with the molds. **(B)** The length and width resolution test results of the patterned electrodes. The coefficients of variation for patterned electrodes with length of 5, 10, 15 mm (A1–A3) were 10.9%, 10.8% and 6.5%, respectively, and the coefficients of variation for patterned electrodes with width of 1.0, 1.5, 2.0 mm (B1–B3) were 6.7%%, 2.1% and 1.4%, respectively. The numbers above the histograms refer to the standard deviations. **(C)** Relationship between the resistance of printed electrodes on silicon with different spin-coating speeds (*N* = 4). **(D)** Relationship between the resistance of printed electrodes on PDMS with different spin-coating speeds (*N* = 4).

To evaluate the resolution and repeatability of the printed patterns by this movable type bioelectronics printing technology, a series of electrodes with length varying between 5 and 15 mm and width between 1.0 and 2.0 mm were patterned and measured (Fig. 3A). The results showed that the coefficients of variation of width and length were no more than 11.0% in our experiments (Fig. 3b). On the other hand, the resistance of transfer printed electrodes using conductive paste prepared at different spin-coating speeds was characterized. Electrode patterns with the length of 5, 10 and 15 mm and the width of 1.5 mm were used for printing. Figure 3C,D show that the resistance of printed electrodes increased as the spin-coating speed increased, where the printed electrodes became thinner. The resistance of printed electrodes also increased with the length of the electrode pattern. These demonstrated that the resistance of transfer printed bioelectronic components could be adjusted by changing spin-coating speed or designing the electrode length or width.

**Figure 4:**
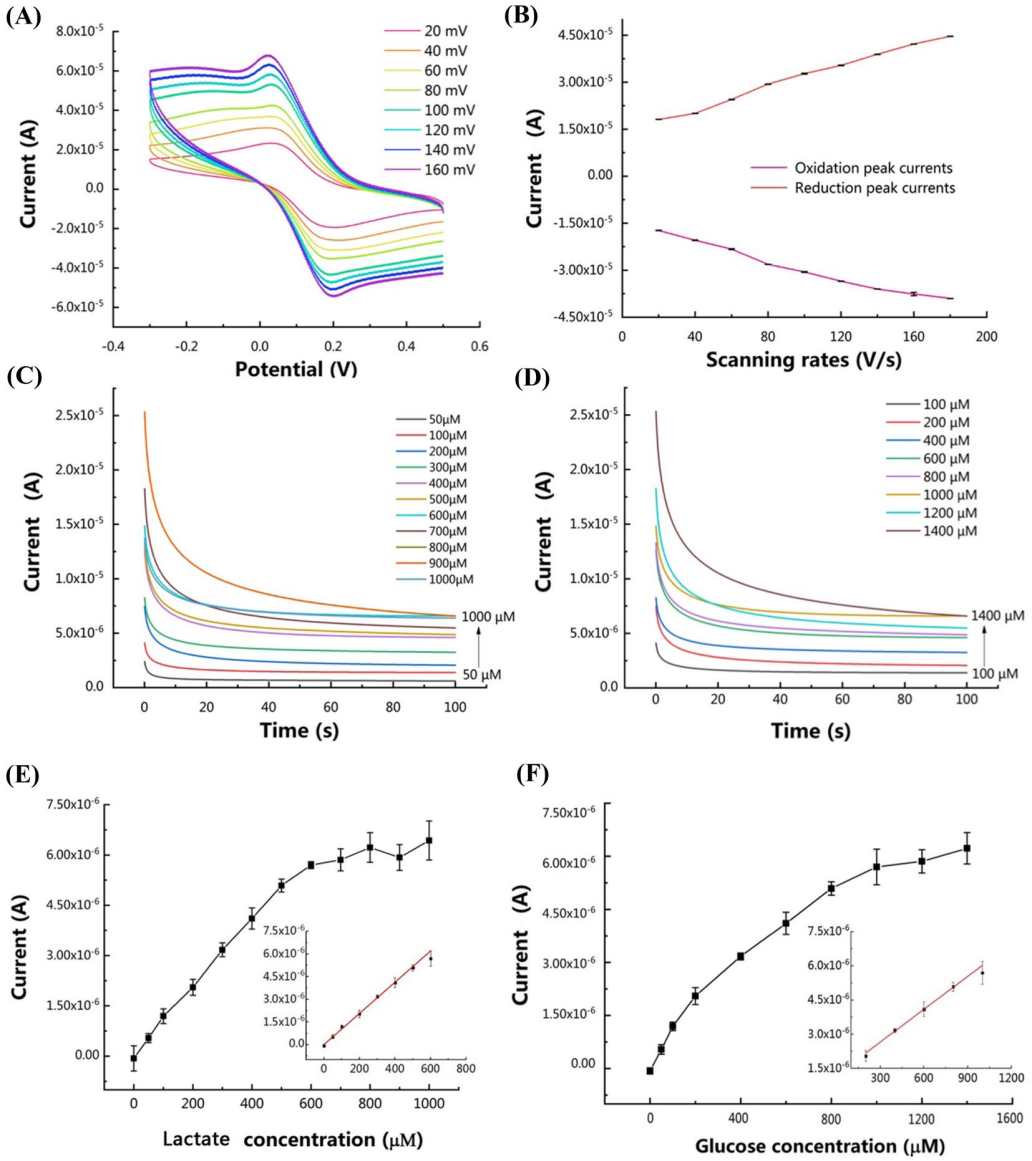
Electrochemical results of the dual-channel biosensor. **(A)** Cyclic voltammetry results of the biosensor in 100 μL potassium ferricyanide solution (0.01 M) under different scanning rates. Scanning rates: 20, 40, 60, 80, 100, 120, 140, 160, 180 mV/s. The oxidation current increased as the scanning rate increased. **(B)** Relationship between peak oxidation and reduction currents versus scanning rates. Error bar was plotted in black (*N* = 3). The peak oxidation current increased as the scanning rate increased in a linear manner. **(C)** Amperometric i–t curve results of the dual-channel biosensor in 0.01 M PBS (pH 7.4) containing lactate with concentrations from 0 to 1000 μM. **(D)** Amperometric i–t curve results of the dual-channel biosensor in 0.01 M PBS (pH 7.4) containing glucose with concentrations from 0 to 1400 μM. **(E)** The relationship between peak oxidation currents and concentrations of lactate (*N* = 4). The insert shows linear curve fitting between lactate concentrations and measured currents, coefficient of association R^2^ = 0.9965 (*N* = 4). The linear range was 0–600 μM and the sensitivity was 103.2 μA/(mM⋅cm^2^) (The area of the working electrode was 0.1 cm^2^). **(F)** The relationship between peak oxidation currents and concentrations of glucose (*N* = 4). The insert shows linear curve fitting between glucose concentrations and measured currents, coefficient of association R^2^ = 0.9941 (*N* = 4). The linear range was 200–1000 μM and the sensitivity was 48.0 μA/(mM⋅cm^2^) (the area of the working electrode was 0.1 cm^2^).

To further demonstrate the capability of this modular transfer printing technology for fabrication of biosensors, a dual-channel electrochemical biosensor for detection of both lactate and glucose was fabricated as shown in Fig. S1. Figure 4A shows the cyclic voltammograms of the dual-channel biosensor under different scanning rates from 20 to 180 mV/s with an interval of 20 mV/s. The peak oxidation current of cyclic voltammetry curves increased in a linear manner as the scanning rate increased (Fig. 4B). This suggests that electron transfer of the dual-channel biosensor was surface adsorption controlled, which means the rate of the REDOX reaction depends on the adsorption rate between the reactants and the active substances on the surface of the working electrode. A comparison test between dual-channel biosensors with or without enzymes in the working electrode was carried out and the one with enzymes exhibited better response for detection of lactate and glucose, which demonstrated outstanding electrocatalytic performance of the transfer printed biosensor with lactate oxidase and glucose oxidase (Fig. S2). Amperometric i-t curves were recorded to further evaluate the sensitivity of the dual-channel biosensor with different concentrations of lactate and glucose as shown in Fig. 4C,D. Figure 4E,F show the peak oxidation currents corresponding to different concentrations of lactate and glucose. As seen from Fig. 4E, the peak oxidation current corresponding to different concentrations of lactate solutions increased steadily from 100 to 800 μM. At concentrations higher than 800 μM, the peak oxidation current reached saturation. Similar phenomenon appeared in Fig. 4F. The insert in Fig. 4E,F showed the linear curve fitting between analyte concentrations and currents. The linear range of the dual-channel biosensor for lactate detection was 0–600 μM and the sensitivity was 103.2 μA/(mM⋅cm^2^), while for glucose detection the linear range was 200–1000 μM and the sensitivity was 48.0 μA/(mM⋅cm^2^). The sensitivity of the dual-channel biosensor was on the same level as other planar biosensors such as paper-based screen-printing biosensors.

**Figure 5:**
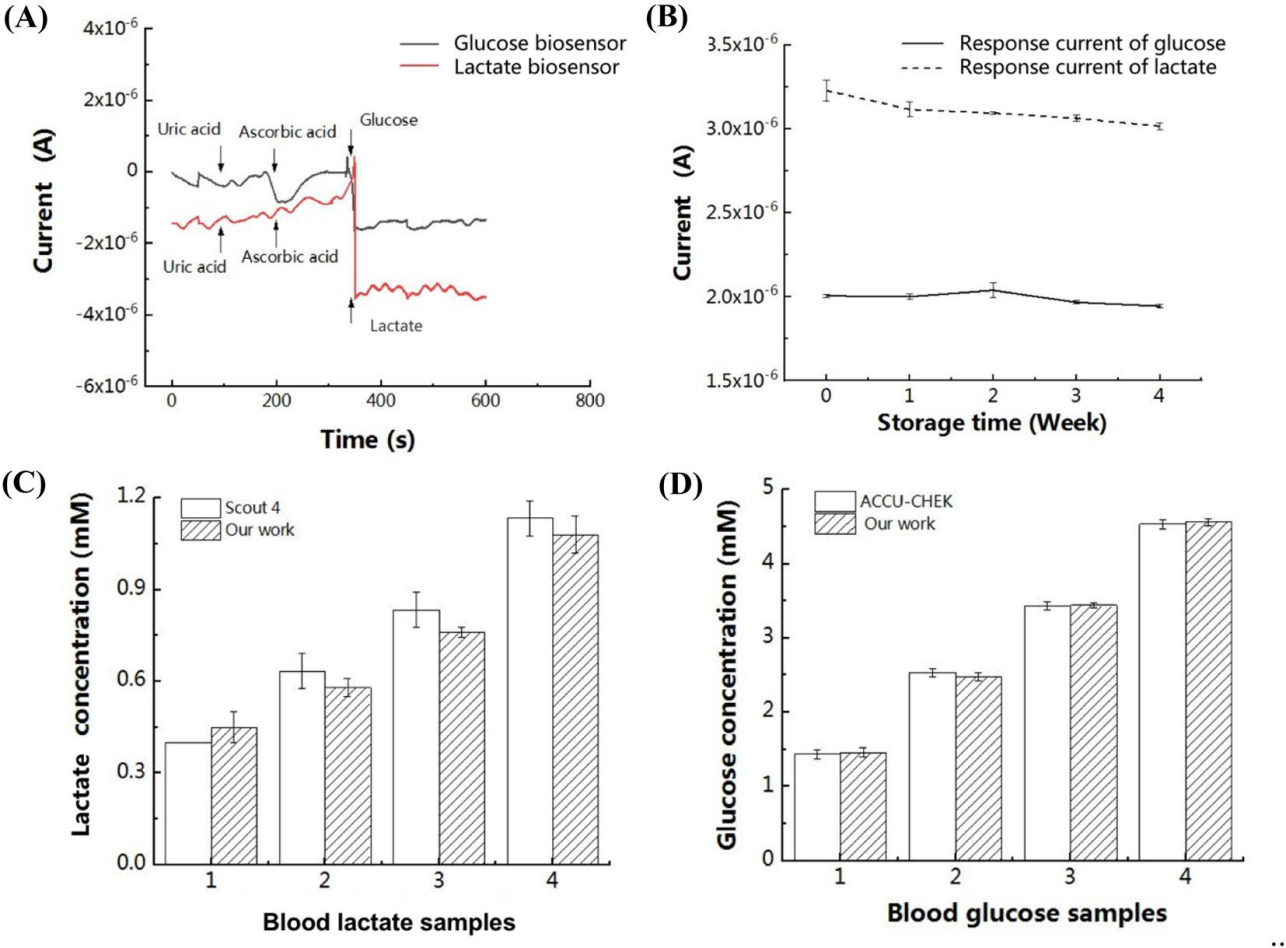
**(A)** Interference tests of the dual-channel biosensor upon addition of uric acid (0.1 mM), ascorbic acid (0.1 mM) in100 μL PBS solution. **(B)** Stability testing results of the dual-channel biosensor in lactate solution with a concentration of 300 μM (dotted line) and glucose solution with a concentration of 300 μM (solid line) after storage in a refrigerator at 4 ˚C after a week. (*N* = 4). **(C)** Concentrations of lactate in real serum samples collected from human measured using our biosensor compared with a Lactate Scout 4 system (*N* = 4, p = 0.328, Paired T-test). **(D)** Concentrations of glucose in real serum samples collected from human measured using our biosensor compared with an ACCU-CHEK analyzer (*N* = 4, p = 0.968, paired T-test).

As seen in Fig. 5A, to study the specificity of the dual-channel biosensor, amperometric i–t measurement of the biosensor was carried out by adding different interferents such as uric acid at 0.1 mM, and ascorbic acid at 0.1 mM, respectively, into 100 μL PBS solution. The concentration of each interferent was chosen based on the physiological concentration level in healthy human blood. The results showed that these interfering species exhibited no effect on the biosensor response, indicating that our fabricated biosensor was feasible for specific detection of lactate and glucose in the presence of other interferents. The fabricated dual-channel biosensor demonstrated good stability by comparing the performance after storage in a 4 ˚C refrigerator for at most 4 weeks to that of the freshly fabricated ones (Fig. 5B). After storage, the response current of the dual-channel biosensor for glucose detection remained steady for the duration of 4 weeks, while for lactate detection the response current slightly decreased with time. The coefficients of variation for glucose and lactate testing of the freshly fabricated dual-channel biosensors were less than 2.08% and 1.94%, respectively. And the coefficients of variation for glucose and lactate testing after 4 weeks storage were 3.09% and 6.81%, respectively. The results showed that the mean response current decreased about 5.7% which might be attributed to the leakage or denaturation of the enzymes. The electrochemical analysis of the catalytic activity of enzymes provided valuable insights of the integrity and functionality of the immobilized enzymes on the biosensor. Still, these results showed that the biosensor maintained good performance for the duration of 4 weeks, which is advantageous compared to other research whose biosensor retained about 91% of the initial current response after 2 weeks^27^. These results suggest that after 4 weeks storage, the dual-channel biosensor could maintain excellent stability for analytical applications with necessary calibration. Besides in the future, we would like to conduct further experiments to test the activity of enzymes using both FTIR ((Fourier Transform Infrared)) and electrochemical methods.

To confirm the feasibility of the developed dual-channel biosensor for detection of target analytes in biological fluids fabricated by the proposed movable type bioelectronics printing technology, serum samples from healthy human volunteers were tested with our dual-channel biosensor and compared with commercial electrochemical analyzers ACCU-CHEK from Roche Diagnostics Corporation for glucose measurement and Lactate Scout 4 from EKF Diagnostics Co., Ltd. for lactate measurement. The results showed no significant difference between lactate and glucose concentrations measured by our dual-channel biosensor and by commercial analyzers (p = 0.328, 0.968 for lactate and glucose, respectively) using Paired T-test (Fig. 5C,D), demonstrating that the accuracy of the developed dual-channel biosensor by movable type printing is sufficient for electrochemical detection in real samples.

From the above results, the capability and feasibility of the proposed movable type bioelectronics printing technology have been successfully demonstrated for the fabrication of biosensors. This simple, low-cost, yet robust printing method could realize direct transfer printing of individual or assembled master molds on rigid or flexible substrates. Additionally, this movable type bioelectronics printing technology could facilitate the fabrication and direct functionalization of bioactive materials.

## Conclusion

In this work, we have successfully developed a movable type bioelectronics printing technology. This movable type bioelectronics printing technology could directly transfer print bioelectronic circuits on both rigid and flexible substrates through the process of biosensor design, mold design and fabrication, conductive material preparation, transfer printing and post-transfer treatment, with high production efficiency, high flexibility and good applicability. Additionally, this movable type bioelectronics printing technology holds advantages in repeatability of pattern transfer, easy modification and flexible combination for fabrication of biosensors, as well as direct fabrication and functionalization with bioactive materials on both rigid and flexible substrates. Finally, biosensors with different functions could be easily implemented by adjusting the conductivity, hydrophobicity or bioactive materials of the prepared paste so as to meet the requirements of different applications. With this new transfer printing technology, we have successfully fabricated electrodes with different geometry and different thickness and studied their characteristics. A dual-channel biosensor for both glucose and lactate detection was fabricated by the movable type bioelectronics printing technology. The results demonstrated that the fabricated dual-channel biosensor exhibited great performance towards both glucose and lactate monitoring.

In summary, the movable type bioelectronics printing technology holds advantages of high repeatability, flexibility and low cost in fabrication of biosensors on rigid and flexible substrates, as well as direct transfer printing with bioactive materials, which helps promote prototyping and small-scale production efficiency of biosensors. We envisage that this modular transfer printing technology might also facilitate the fabrication of a wide range of bioelectronic components and sensors for development of wearable devices.

## Supplementary Information

Supplementary Information.
