# Supplementary material for: A movable type bioelectronics printing technology for modular fabrication of biosensors
Source: Sci Rep. 2021 Nov 16;11:22323. doi: 10.1038/s41598-021-01741-1 (PMC8595314; doi:10.1038/s41598-021-01741-1)
Supplement: Supplementary file 1 — Supplementary Information. [file 41598_2021_1741_MOESM1_ESM.docx]

Supplementary Materials for

A movable type bioelectronics printing technology for modular fabrication of biosensors

Muqun Yang^1,2†^, Mingyang Liu^1,2^, Jing Cheng^1,2*^, Han Wang^2*†^

^1^Precision Medicine and Healthcare Research Center, Tsinghua-Berkeley Shenzhen Institute (TBSI), Tsinghua University, Shenzhen, 518055, China
^2^Department of Biomedical Engineering, School of Medicine, Tsinghua University, Beijing, 100084, China

^†^These authors contributed equally to this work.
^*^Corresponding authors: jcheng@tsinghua.edu.cn; hanwang@tsinghua.edu.cn

**.**


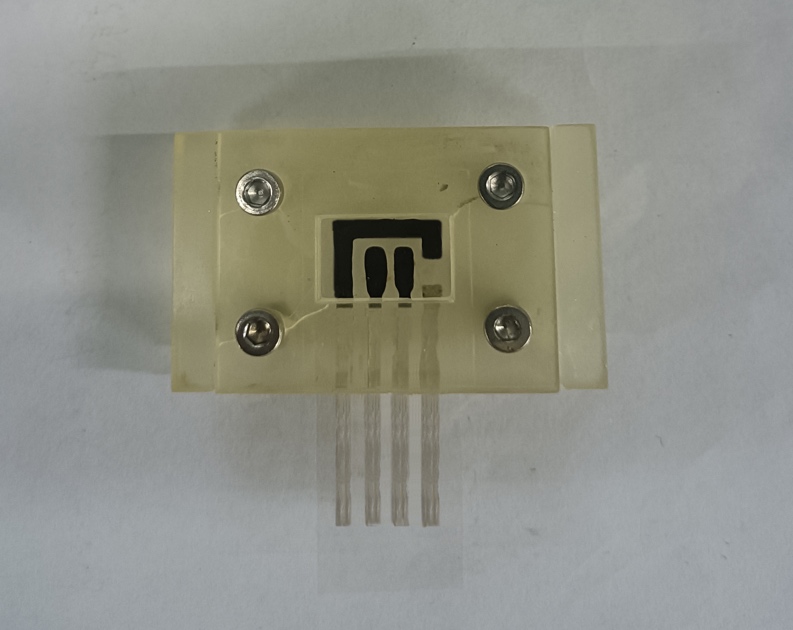


**Fig. S1** Photographic image of the printed dual-channel biosensor using movable type bioelectronics printing technology.


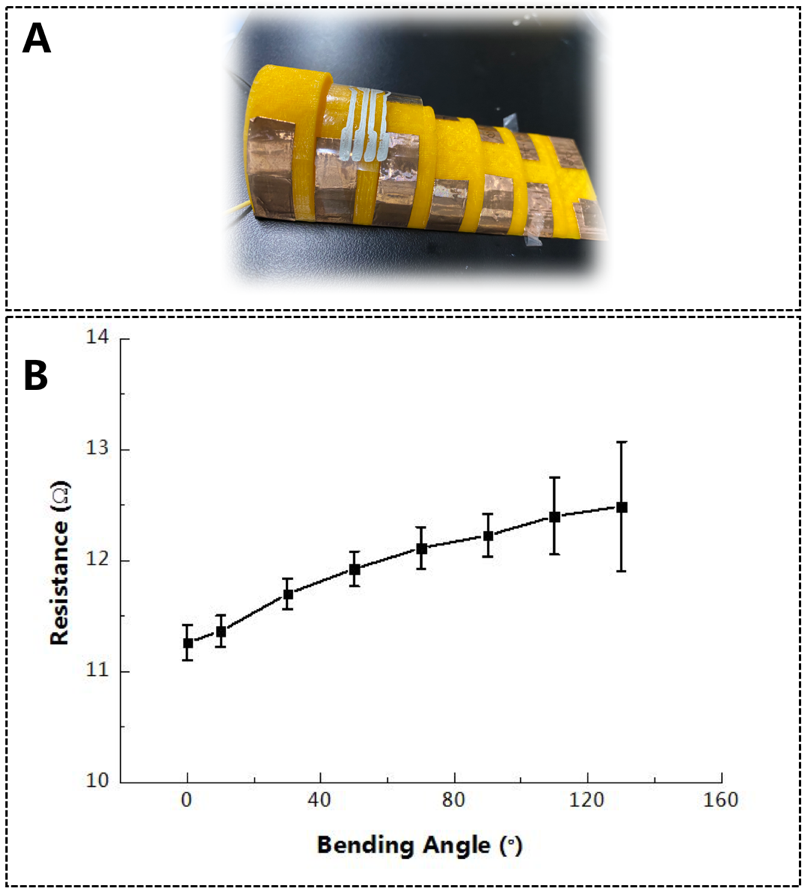


**Fig. S2** The resistance changes of the printed electrodes after being bent at different angles. (A) Experimental setup for measurement of the resistance of transfer printed electrodes after bending. (B) Relationship between the resistance of the transfer printed electrodes and the bending angles. (*N* = 5)

Table S1. Spin coating speed for the printed electrodes

| **Low speed** | **Time 1** | **High speed** | **Time 2** |
| --- | --- | --- | --- |
| 1000 rpm | 20 s | 1000 rpm | 40 s |
| 1000 rpm | 20 s | 1500 rpm | 40s |
| 1000 rpm | 20 s | 2000 rpm | 40s |
| 1000 rpm | 20 s | 2500 rpm | 40s |
| 1000 rpm | 20 s | 3000 rpm | 40s |

Table S2. Length resolution test of the printed electrodes.

| **Length resolution test** | | | | | | | | | | |
| --- | --- | --- | --- | --- | --- | --- | --- | --- | --- | --- |
| **Pattern 1** | **Pattern 2** | **Pattern 3** | **Pattern 4** | **Pattern 5** | **Pattern 6** | **Pattern 7** | **Pattern 8** | **Average /mm** | **SD** | **CV** |
| 5.20 | 5.10 | 6.00 | 5.50 | 5.20 | 5.50 | 6.00 | 6.00 | **5.20** |  | **6.70%** |
| 5.00 | 5.10 | 5.10 | 4.80 | 5.00 | 5.00 | 5.00 | 5.10 |  | **0.35** |  |
| 4.90 | 5.05 | 5.10 | 4.80 | 5.10 | 5.10 | 5.10 | 5.00 |  |  |  |
| 10.10 | 10.50 | 10.50 | 10.10 | 10.10 | 10.50 | 10.70 | 10.50 | **10.19** | **0.21** | **2.10%** |
| 10.05 | 10.50 | 10.10 | 10.10 | 10.10 | 10.00 | 10.10 | 10.00 |  |  |  |
| 9.90 | 10.10 | 10.20 | 10.05 | 10.10 | 10.10 | 10.00 | 10.10 |  |  |  |
| 15.50 | 15.60 | 15.50 | 15.50 | 15.20 | 15.00 | 15.50 | 15.50 | **15.27** | **0.21** | **1.40%** |
| 15.10 | 15.50 | 15.10 | 15.10 | 15.10 | 15.10 | 15.50 | 15.10 |  |  |  |
| 15.40 | 15.10 | 15.10 | 15.10 | 15.20 | 15.00 | 15.50 | 15.20 |  |  |  |

Table S3. Width resolution test of the printed electrodes.

| **Width resolution test** | | | | | | | | | | |
| --- | --- | --- | --- | --- | --- | --- | --- | --- | --- | --- |
| **Pattern 1** | **Pattern 2** | **Pattern 3** | **Pattern 4** | **Pattern 5** | **Pattern 6** | **Pattern 7** | **Pattern 8** | **Average /mm** | **SD** | **CV** |
| 1.05 | 1.40 | 1.10 | 1.20 | 1.50 | 1.40 | 1.50 | 1.30 | **1.29** | **0.14** | **10.90%** |
| 1.10 | 1.30 | 1.10 | 1.50 | 1.40 | 1.30 | 1.30 | 1.30 |  |  |  |
| 1.20 | 1.30 | 1.10 | 1.20 | 1.50 | 1.30 | 1.30 | 1.20 |  |  |  |
| 1.10 | 1.60 | 1.55 | 1.40 | 1.50 | 1.70 | 1.60 | 1.50 | **1.57** | **0.17** | **10.80%** |
| 1.20 | 1.60 | 1.70 | 1.50 | 1.50 | 1.50 | 1.75 | 1.60 |  |  |  |
| 1.30 | 1.70 | 1.70 | 1.75 | 1.70 | 1.60 | 1.75 | 1.75 |  |  |  |
| 2.05 | 2.00 | 2.10 | 2.10 | 2.50 | 2.20 | 2.30 | 2.10 | **2.16** | **0.14** | **6.50%** |
| 2.05 | 2.10 | 2.10 | 2.10 | 2.40 | 2.10 | 2.10 | 2.00 |  |  |  |
| 2.10 | 2.10 | 2.05 | 2.10 | 2.10 | 2.20 | 2.40 | 2.40 |  |  |  |


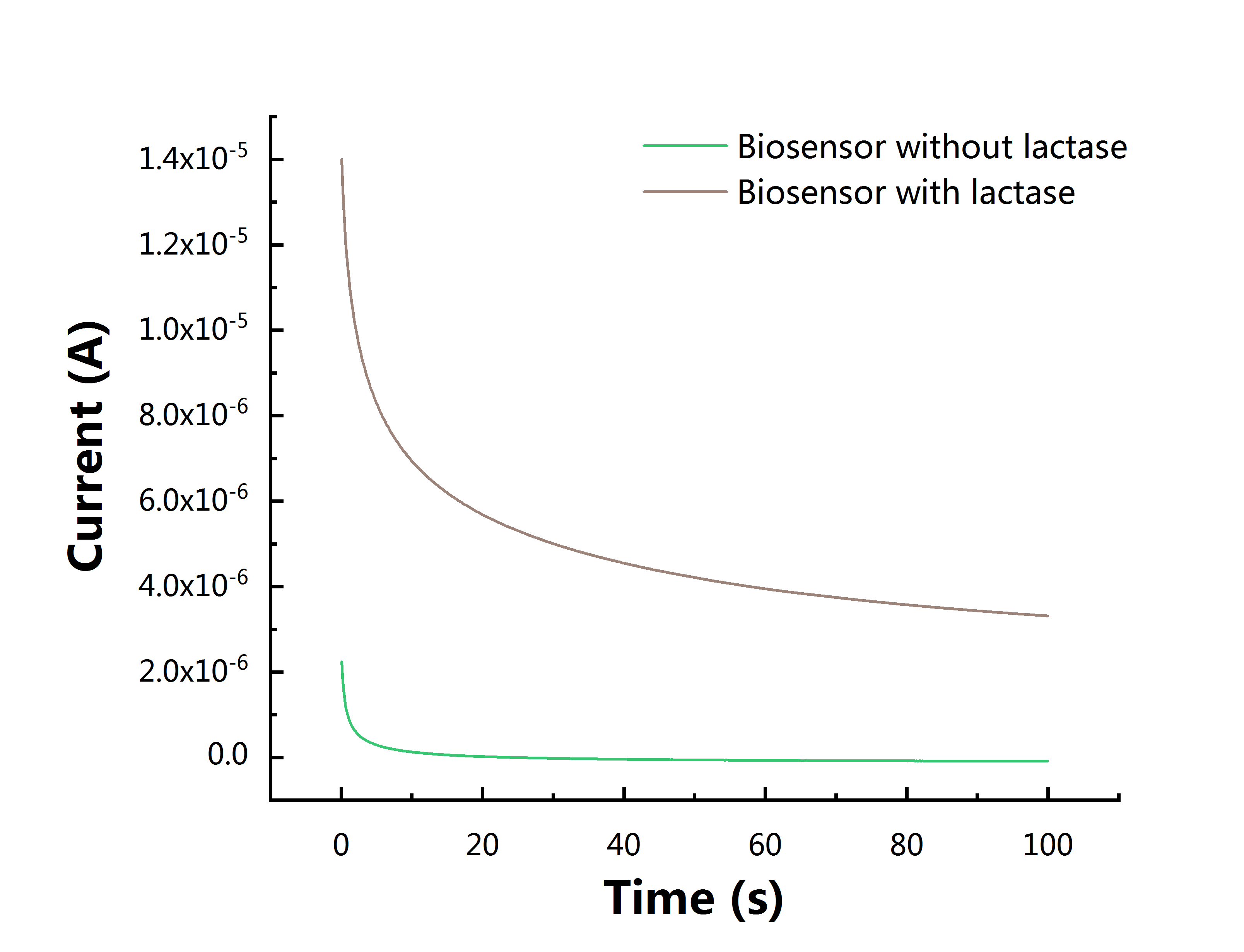

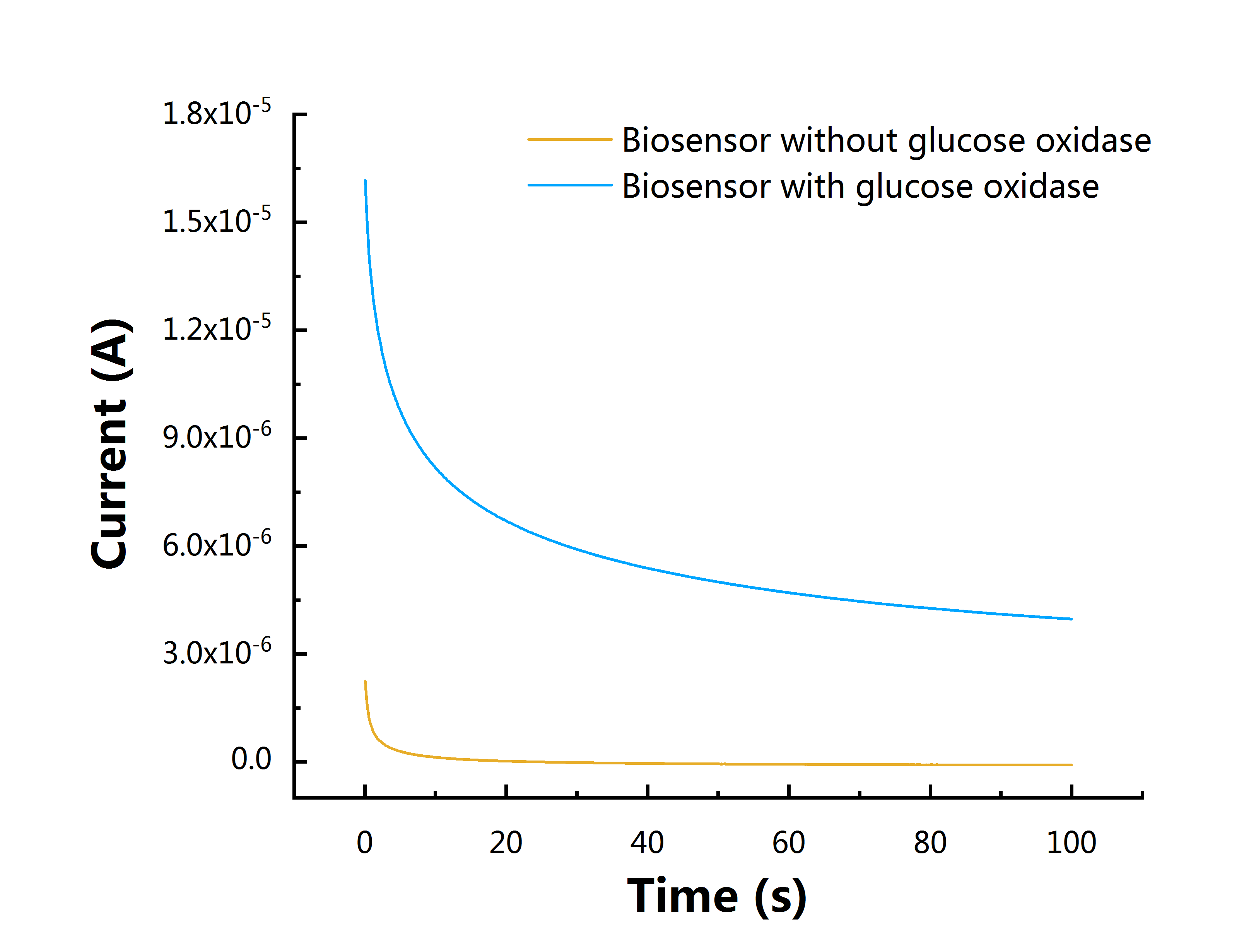


**Figure. S3** Amperometric i-t curve response of the dual-channel biosensor in PBS (pH=7.4) containing 300 $\mu$M lactic acid and glucose. The potential of amperometric i-t tests was set at 0.30 V.
